# Solvent-Free Lipid Separation and Attenuated Total Reflectance Infrared Spectroscopy for Fast and Green Fatty Acid Profiling of Human Milk

**DOI:** 10.3390/foods11233906

**Published:** 2022-12-03

**Authors:** Christopher Karim Akhgar, Victoria Ramos-Garcia, Vanessa Nürnberger, Alba Moreno-Giménez, Julia Kuligowski, Erwin Rosenberg, Andreas Schwaighofer, Bernhard Lendl

**Affiliations:** 1 Institute of Chemical Technologies and Analytics, Technische Universität Wien, Getreidemarkt 9, 1060 Vienna, Austria; 2 Health Research Institute La Fe, Avenida Fernando Abril Martorell 106, 46026 Valencia, Spain; 3 Competence Center CHASE GmbH, Altenberger Straße 69, 4040 Linz, Austria

**Keywords:** mid-infrared spectroscopy, attenuated total reflection, human milk, fatty acids, green analytical chemistry, partial least squares

## Abstract

This study presents the first mid-infrared (IR)-based method capable of simultaneously predicting concentrations of individual fatty acids (FAs) and relevant sum parameters in human milk (HM). Representative fat fractions of 50 HM samples were obtained by rapid, two-step centrifugation and subsequently measured with attenuated total reflection IR spectroscopy. Partial least squares models were compiled for the acquired IR spectra with gas chromatography-mass spectrometry (GC-MS) reference data. External validation showed good results particularly for the most important FA sum parameters and the following individual FAs: C12:0 (R^2^_P_ = 0.96), C16:0 (R^2^_P_ = 0.88), C18:1cis (R^2^_P_ = 0.92), and C18:2cis (R^2^_P_ = 0.92). Based on the obtained results, the effect of different clinical parameters on the HM FA profile was investigated, indicating a change of certain sum parameters over the course of lactation. Finally, assessment of the method’s greenness revealed clear superiority compared to GC-MS methods. The reported method thus represents a high-throughput, green alternative to resource-intensive established techniques.

## 1. Introduction

Human milk (HM) is a complex, dynamic, and bioactive fluid with the fundamental role of supplying infants with all essential nutrients before they are able to digest solid food [1]. According to the World Health Organization (WHO), exclusive breastfeeding is recommended for the first six months of life, followed by complementary breastfeeding of up to two years or beyond [2]. The HM macronutrient composition is known to be significantly influenced by factors such as prematurity or postnatal age in order to fulfil the nutritional demands across all stages of development [3]. In this context, the total protein concentration significantly decreases, whereas fat and lactose content increase during the first weeks of lactation, before average concentrations of 1.0 g/dL protein, 3.4 g/dL fat and 6.7 g/dL lactose are reached for mature HM (>2 weeks postpartum) [4].

HM fat occurs as milk fat globules emulsified in water, consisting of a milk fat globule membrane that envelopes a core rich in triglycerides (TAGs). TAGs, consisting of a glycerol backbone and three fatty acids (FAs), account for approximately 98% of total HM lipids [5]. The FA composition is the most variable macronutrient, known to be significantly influenced by factors such as mothers’ genetics and diet, obesity, stage of lactation, socio-demographic variables, and the environment [6,7]. In this context, it has been demonstrated that the HM FA composition varies significantly between mothers with omnivore, vegan, and vegetarian diets [8]. Furthermore, the FA profile of HM from European mothers with western diet differs in the amount of saturated FAs (SAT), monounsaturated FAs (MONO), and polyunsaturated FAs (PUFA) from lactating mothers with other forms of diet [9]. With the progress of lactation, the relative content of medium-chain FAs (MCFA, C11-C16,) increases, whereas long-chain FAs (LCFA, C17 and higher) and PUFAs decrease [10,11].

When not enough of one’s own mother’s milk is available, pasteurized donor HM (DHM) is preferred over infant formula for feeding of preterm infants, offering immunological advantages and lower rates of necrotising enterocolitis [12]. The established method to destroy vegetative bacteria and viruses in DHM, thus ensuring safety for the infants, is called “Holder” pasteurization (HoP), where samples are heated to 62.5 °C for 30 min [13]. The effect of HoP on HM lipids is still a topic of discussion, as available studies report different results, ranging from no influence to a decrease of total fat content and certain FAs [14,15]. Consequently, reliable analytical methods for monitoring the FA profile of DHM are of major interest.

Established methods for determining the FA profile in HM are based on gas chromatography (GC) with different detectors such as flame ionization detectors (FID) or mass spectrometry (MS) [15,16]. These methods offer excellent sensitivity and accuracy, but bear severe drawbacks such as expensive instrumentation, time intensive chromatographic runs of approximately 1–2 h, and a labour-intensive derivatization step prior to analysis, typically involving hazardous chemicals. Particularly, in the context of green analytical chemistry (GAC), there is a growing demand for more time- and energy-efficient methods that do not involve toxic substances or produce large amounts of waste [17].

Mid-infrared (IR) spectroscopy is capable of providing qualitative and quantitative information about clinically relevant parameters by detecting absorption of IR radiation through fundamental molecular vibrations [18]. Fourier-transform infrared (FTIR) instruments are nowadays considered as the gold-standard, offering rapid spectra acquisition and high accuracy while covering the whole mid-IR region (400–4000 cm^−1^). Compared to GC-MS, this technique offers advantages in terms of reduced measurement times, lower cost, minimum to no sample preparation, and non-destructive operation. Commercially available mid-IR analyzers, operating in transmission configuration, tailored for HM analysis (e.g., Miris Ab Human Milk Analyzer (HMA), Sweden) allow for determination of the HM macronutrient composition without the need for specialized staff or expensive laboratory facilities. It has been demonstrated that results for total protein, fat, and lactose content obtained with these analysers are widely acceptable [19]. As an alternative to transmission measurements, attenuated total reflection (ATR) is often applied as a more robust probing mode. Here, the IR beam is totally reflected at the interface between an optically denser ATR crystal and the optically rarer sample, leading to the formation of an evanescent wave that can interact with the sample at typical penetration depths of 1–2 µm [20].

Predicting the FA composition of milk from different mammals by IR spectroscopy is usually performed by relating the mid-IR transmission spectra of whole milk to GC reference concentrations by calculation of multivariate partial least squares (PLS) regression models [21,22]. This approach, however, suffers from the overlap of the target TAG bands with large absorbance bands of water, proteins, and lactose in whole milk transmission spectra. Consequently, the obtained results are dominated by cross-correlations between individual FA concentrations and total fat content [23]. In this context, it has been demonstrated that fat separation prior to mid-IR spectra acquisition is highly beneficial, as in this way, cross-correlations between individual FAs and total fat are inherently eliminated and FA-related features are better resolved than for whole milk spectra [24]. Most recently, a solvent-free lipid separation method [25] was combined with attenuated total reflection Fourier-transform infrared (ATR-FTIR) spectroscopy to show the proof-of-concept for mid-IR based FA profiling in HM based on a limited data set of eight samples [26]. Here, good PLS models were obtained for some important FA sum parameters such as unsaturated FAs (UNSAT), SAT, MCFA, and LCFA.

In the present work, the developed approach was expanded to a larger data set, involving unprocessed DHM samples, as well as samples that underwent HoP. A comprehensive method validation based on splitting of the available samples into a training and a validation set was performed, showing very good prediction results for the most important sum parameters as well as certain individual FAs. Subsequently, the obtained PLS models were applied to investigate the effect of different clinical parameters including the stage of lactation, birth weight, infant’s sex, gestational age, maternal age, and HoP on the HM FA profile, which was previously only possible by time- and labour-intensive GC methods. Finally, the greenness of the method was demonstrated based on a comparison to GC-MS reference methods by applying the Analytical GREEnness (AGREE) metric approach [27].

## 2. Material and Methods

### 2.1. Human Milk Samples

The study was approved by the Ethics Committee for Biomedical Research of the Health Research Institute La Fe, University and Polytechnic Hospital La Fe (Valencia, Spain) with the approval number 2019-289-1. All methods were conducted according to current guidelines and regulations. Healthy donors were admitted to participation after routine screening and interview by the Human Milk Bank at the University and Polytechnic Hospital La Fe and after providing written informed consent.

Human milk samples (*n* = 50) with volumes ranging between 50 and 200 mL were collected corresponding to the guidelines of the hospital staff. Milk expression was performed using breast milk pumps in accordance with the standard operating procedure implemented at the hospital and the Human Milk Bank. Removable parts of the collection, bottles and breast milk pumps, were sterilized before milk extraction. Furthermore, donors had to wash their hands with soap and water and the skin area that comes in contact with the milk pump with water. HM was extracted at the participants’ homes and stored at −20 °C before being transported to the Human Milk Bank. Extracted HM (*n* = 26) was stored at −20 °C until further processing. The remaining HM samples (*n* = 24) were pooled HM samples obtained by mixing several aliquots extracted at different days from the same donor, which then underwent HoP prior to storage at −20 °C until further processing. Data including maternal age, infant’s postnatal age, infant’s sex, infant’s birth weight, and gestational age were collected.

### 2.2. Macronutrient Analysis

Human milk macronutrients (i.e., crude and true protein, carbohydrate, fat, total solids, and energy) were analyzed employing the MIRIS HMA™ (MIRIS AB, Uppsala, Sweden). Instrument calibration, quality control, and measurements were conducted according to the manufacturer’s standard operation procedure.

### 2.3. Solvent-Free Lipid Separation

HM lipid separation was performed according to a previously proposed [28] and later modified [25] solvent-free centrifugation approach. Here, 30 mL HM sample aliquots were thawed overnight at 4 °C, followed by tempering at room temperature for at least 20 min. Subsequently, samples were centrifuged for 30 min at 17,800× *g* and 20 °C in a Sigma 3–18 k centrifuge (Sigma Laborzentrifugen GmbH, Osterode am Harz, Germany). The upper cake was transferred into microtubes and centrifuged for 20 min at 19,300× *g* at the same temperature. The obtained upper layer, consisting of pure HM lipids, was removed and used for ATR-FTIR and GC-MS measurements.

### 2.4. ATR-FTIR Measurements

FTIR absorption measurements of HM lipids were performed at room temperature using a Bruker Tensor 37 FTIR spectrometer (Ettlingen, Germany) equipped with a Bruker Optics Platinum ATR module and a liquid nitrogen cooled HgCdTe (mercury cadmium telluride) detector. IR spectra of one drop of pure HM fat were acquired in the range from 600 to 4000 cm^−1^ with a spectral resolution of 2 cm^−1^ in a double-sided acquisition mode. A total of 128 scans (52 s acquisition time) were averaged per spectrum, which was calculated using a Blackman-Harris 3-term apodization function and a zero-filling factor of 2. After each HM fat measurement, the ATR crystal was cleaned with Glucopon 600 CS UP solution (~50% in water, Sigma-Aldrich, Steinheim, Germany) and water to recover the initial baseline signal. In order to reduce the influence of water vapor from the atmosphere on the recorded spectra, the instrument was constantly flushed with dry air. Spectra were acquired and analyzed using the software package OPUS 8.1 (Bruker, Ettlingen, Germany).

### 2.5. GC-MS Reference Measurements

For GC-MS reference measurements, solutions of 20 mg/mL pure HM fat in dichloromethane were prepared. In total, 50 µL of these solutions were transferred into cooled glass vials with micro inserts and mixed with 50 µL internal standard (0.2 mg/mL C15:0 in dichloromethane) and 50 µL of a trimethylsulfonium hydroxide (TMSH, 0.25 M in MeOH, Supelco, Bellefonte, PA, United States) derivatization agent. Subsequently, vials were immediately capped and vortexed for 5 s, following by heating to 70 °C for 15 min in order to complete the derivatization reaction.

A Shimadzu GC-2010 Plus Chromatography system coupled to a single-quadrupole mass spectrometer (Shimadzu, Kyoto, Japan), equipped with a ZB-FAME column (30 m, 0.25 mm I.D., 0.20 µm film thickness; Phenomenex, Aschaffenburg, Germany), was used for all GC-MS measurements. A total of 1 µL of the derivatized solutions were injected in split mode using a split of 100:1, by a Shimadzu AOC-5000 Plus autosampler. The injector temperature was set to 250 °C. A constant septum flow rate of 3 mL/min was applied with a column flow rate of 2.14 mL/min. The initial oven temperature of 40 °C was held for 3 min, then increased by 10 °C per minute to 100 °C, and further increased by 2 °C per minute to 200 °C. Ion source and transferline temperature were both kept constant at 200 °C. After a solvent vent of 2.7 min, data acquisition was performed in scan mode (*m*/*z* 35-500) with a detector voltage of 1.05 kV.

For quantitative analysis, calibration was performed by preparing appropriate dilutions of a 37 component FAME mix certified reference material (TraceCERT^®^, Supelco, Bellefonte, PA, United States). Quantitative evaluation was performed based on the quantifier ion peak area for each FAME, if the ratio of quantifier and qualifier ions was within acceptable thresholds. Retention times, qualifier, and quantifier ions for each analyte are reported elsewhere [24]. The results of GC-MS analysis of all HM milk samples are shown in Tables S1–S4.

### 2.6. Data Analysis

PLS modelling and principal component analysis (PCA) were performed in Matlab R2020a (Mathworks Inc., Nattick, MA, USA), using the PLS Toolbox 8.9 (Eigenvector Research Inc., Wenatchee, WA, USA). All ATR-FTIR spectra of pure HM lipids were first pre-processed by mean centering and calculation of 2nd derivative spectra, using a 2nd order polynomial Savitzky-Golay filter with a window of 15 points. For each target parameter, the included spectral region was individually selected based on the PLS selectivity ratio (SR) [29]. First, calibration equations were calculated based on a training set (*n* = 35) and evaluated with contiguous blocks cross-validation with 10 data splits. Second, validation was performed by using these calibrations to predict the concentrations of an external validation set (*n* = 15) and calculating characteristic figures of merit. The samples were randomly assigned to the training and validation sets respectively, so that both HM and HM HoP samples were evenly distributed between sets.

## 3. Results and Discussion

### 3.1. Macronutrient Analysis

A set of 50 HM samples, including 26 HM samples collected prior to HoP and 24 HM samples collected post-pasteurization, was used in this study. The median (interquartile range, IQR) birth weight was 2911 (786) g, whereas maternal, gestational, and postnatal ages were 35 (6) years, 39 + 2 (2 + 2) weeks + days, and 158 (213) days, respectively. A total of 25 HM samples were from mothers of female infants. The macronutrient concentration was determined by mid-IR based Miris HMA™, showing median (IQR) results of 8.0 (0.2) g/dL for carbohydrates, 1.8 (0.8) g/dL for total fat, 0.6 (0.5) g/dL for crude protein, 0.4 (0.4) g/dL for true protein, 10.5 (0.7) g/dL for total solids, and 51 (7) kCal/dL for energy. Compared to the macronutrient composition of mature HM from the literature, the obtained results for carbohydrates are slightly higher, whereas fat and protein concentrations are lower [4]. This study relies on the use of pooled DHM as provided by regular donors enrolled at the hospital HM bank. Although this procedure allows for the study of samples that are similar to those administered at the hospital, the study protocol did not control for clinical variables possibly affecting the macronutrient composition of HM, such as postnatal age and gestational age (term or preterm delivery), or variables related to sample collection such as expression type, time after last feeding/expression, and time of the day [15].

### 3.2. ATR-FTIR Spectra of Separated HM Fat Fraction

**Figure 1:**
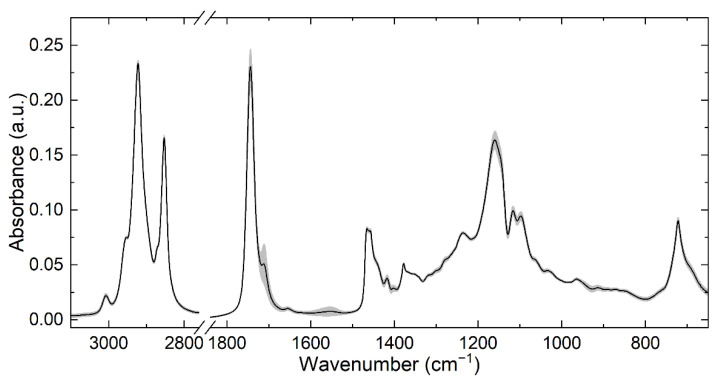
Mean spectrum (black line) of the recorded ATR-FTIR spectra of the separated lipid fraction of HM and variance (1σ) within the dataset of 50 samples (grey area).

TAGs show characteristic absorbance bands in the mid-IR region that are influenced by factors such as FA degree of saturation and chain length [30,31]. Milk, however, is a highly complex matrix with various IR absorbance signals originating from other major components such as water, proteins, and lactose that overlap with the significantly smaller bands from TAGs and that adversely affect their evaluation [24,32]. Consequently, in this work, a representative part of the lipid fraction was isolated from HM samples by a solvent-free centrifugation method, before measuring the purified lipid fraction with ATR-FTIR spectroscopy. Figure 1 shows the mean of recorded ATR-FTIR spectra of the lipid fraction separated from HM samples.

Grey areas indicate the variance (1σ) obtained in the HM fat spectra of the entire data set. The most prominent bands can be attributed to C-H stretching (2850–3000 cm^−1^), C=O stretching (1700–1750 cm^−1^), C-H deformation (1200–1470 cm^−1^, 720 cm^−1^), and C-O stretching (1160 cm^−1^) vibrations [33]. The bands at 1710 cm^−1^ and 1570 cm^−1^ have been attributed to free fatty acids [34].

### 3.3. Mid-IR-Based Determination of the Fatty Acid Profile

Individual PLS calibration models were calculated for each target parameter by relating the recorded ATR-FTIR spectra to GC-MS reference concentrations. The pre-processing step combined mean centering and calculation of second derivatives for all spectra [24]. The recorded ATR-FTIR spectra comprised the entire available mid-IR range (600–4000 cm^−1^); however, the wavenumber range incorporated in the PLS analysis was individually selected for each target parameter based on the PLS selectivity ratio (SR). This is a powerful tool for visualizing features of mid-IR spectra with high correlation to the predicted parameter. In brief, it can be defined as the ratio between explained and unexplained variance for each variable in the data set [29]. Selected wavenumber regions for the applied PLS models, as well as their SR, are shown in Figure S1.

Table 1 shows the obtained statistical parameters for each PLS model. The presented approach predicts relative FA concentrations in g/100 g fat, which inherently eliminates previous problems regarding covariation structures in mid-IR based FA profiling [23]. A training set of 35 HM samples was used to calculate calibration models including typical figures of merit, such as the root mean square error of calibration (RMSEC) and the calibration coefficient of determination (R^2^). Mathematical descriptions of all applied figures of merit are collected in the supplementary information. For internal validation, a contiguous blocks cross-validation with 10 data splits was performed, and the root mean square error of cross-validation (RMSECV) and cross-validation coefficient of determination (R^2^_CV_) were determined. The applied number of latent variables (LVs) was individually selected for each parameter by plotting RMSEC and RMSECV versus the number of LVs (see Figures S2 and S3). Here, the optimum number of LVs was found to be between two and six, which is reasonable for a highly complex mixture such as milk fat, containing a large number of different components that can influence the spectra [24].

**Figure 2:**
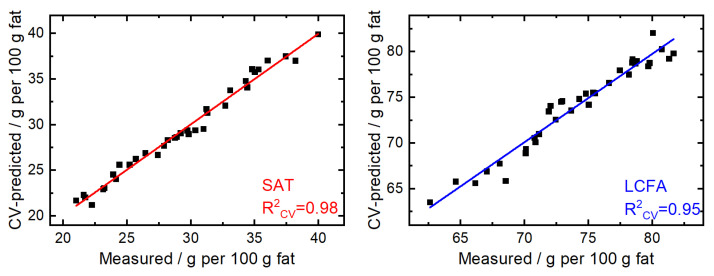
Measured (GC-MS) vs. predicted (ATR-FTIR, cross-validation) fatty acid concentration for the sum parameters of saturated fatty acids (SAT, **left**) and long-chain fatty acids (LCFA, **right**).

Figure 2 shows the relationship between GC-MS reference concentrations and ATR-FTIR based predictions of PLS cross-validation on the examples of SAT and LCFA. Under ideal conditions, all points would fall on the straight regression line, whereas higher and lower points indicate over- and underestimation of mid-IR measurements compared to the reference values. In the present case, the high R^2^_CV_ values of 0.98 and 0.95 as well as the small deviations of data points from the regression lines indicate excellent performance of the models. Relationships for the other PLS models can be found in Figure S4. For a more comprehensive validation, the calculated PLS models were used to determine the concentrations of an external validation set, comprising 15 HM samples. In order to evaluate the accuracy of the predicted concentrations, the root mean square error of prediction (RMSEP) and the prediction coefficient of determination (R^2^_P_) were calculated. Here, excellent results were obtained for the sum parameters SAT (R^2^_P_ = 0.97), MONO (R^2^_P_ = 0.94), PUFA (R^2^_P_ = 0.93), and UNSAT (R^2^_P_ = 0.97) that provide information on the degree of saturation and are of particular interest as their concentration changes depending on the mother’s diet [9]. Furthermore, excellent results were obtained for MCFA (R^2^_P_ = 0.94) and LCFA (R^2^_P_ = 0.94) that provide information regarding FA chain-length and these sum parameters are of special concern as their concentrations change with the stage of lactation [10,11]. Results for the group of short-chain fatty acids (SCFA, C10 and lower, R^2^_P_ = 0.25) were significantly poorer, which is likely based on the fact that the concentration of these FAs is very low in HM. Regarding the prediction of individual FAs, excellent results were obtained for C12:0, C18:1cis, and C18:2cis (R^2^_P_ ≥ 0.92), whereas moderate predictions were obtained for C14:0, C16:0, and C18:0 (R^2^_P_ ≥ 0.71). It should be noted that particularly good prediction results were obtained for the two most abundant cis UNSAT; however, the results for C16:1cis were significantly poorer (R^2^_P_ = 0.19), which is likely based on the lower concentration of this FA which dropped below the limit of quantitation of the GC-MS reference method for certain samples. Consequently, approx. 90–95% of fatty acids in human milk detectable with the employed GC-MS method could be precisely predicted (R^2^_CV_ > 0.84, R^2^_P_ > 0.71) using ATR-FTIR spectroscopy. Finally, the measured and predicted FA profiles were compared by PCA. The loading plots in Figure S5 successfully demonstrates the similarity between the two data sets.

The number of available samples can significantly influence the quality of PLS models. In the present study, the number of samples was noticeably increased compared to a previous study, where the herein applied approach for HM FA analysis was developed and tested [26]. Compared with these previous results, the PLS models for SAT, MONO, PUFA, UNSAT, and LCFA could be clearly improved. Furthermore, it was previously not possible to determine the concentrations of individual FAs. In the present work, some of the most abundant FAs were predicted with high accuracy, consequently representing the first mid-IR based approach capable of determining the most important FA sum parameters and individual FAs in HM simultaneously. 

### 3.4. Investigation of Clinically Relevant Parameters

**Figure 3:**
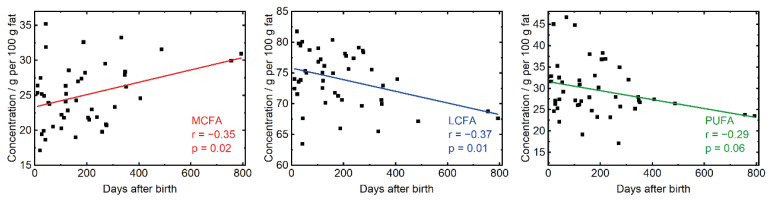
Concentration of medium-chain fatty acids (MCFA), long-chain fatty acids (LCFA), and polyunsaturated fatty acids (PUFA) determined with ATR-FTIR spectroscopy over the course of lactation.

The presented method based on ATR-FTIR spectroscopy was applied to investigate possible effects of clinical parameters on the HM FA profile. For this purpose, the PLS models from Table 1 were employed to predict the FA profile in all available milk samples. First, changes in the FA profile over the course of lactation were monitored by plotting the obtained concentrations against the days after delivery. Figure 3 shows the results for MCFA, LCFA, and PUFA, as these sum parameters were previously found to significantly change over the course of lactation in a meta study with pooled data analysis [11]. The results for the remaining sum parameters are shown in Figure S6. The linear fit, as well as the *p*-value (two-tailed) of 0.02 for MCFA, indicates a significant increase of this sum parameter over the course of lactation. These findings agree well with the pooled data analysis [11]. Furthermore, Figure 3 shows a significant decrease of LCFA with progressing lactation, which also was found in the above-mentioned meta-study [11]. Finally, the herein observed reduction of PUFA was also reported in the meta-study.

In the next step, the FA profile was compared between HM samples prior to HoP and post-pasteurization. The boxplots in Figure S7 show no significant differences for the most important FA sum parameters in pre- and post-pasteurized DHM. These results agree well with most previous studies that investigated the effect of HoP on the relative HM FA profile reporting no significant difference [35,36,37,38]. In a recent study based on direct derivatization of FAs from HM and subsequent GC-MS analysis, a small but significant decrease of certain absolute FA concentrations (particularly SAT, LCFA, MONO, PUFA) was found following HoP [15]. However, the design of this study involved the analysis of paired pre- and post-pasteurization DHM samples, which eliminates the biological background variation that might exist between donors and samples and therefore allows for a more sensitive evaluation of changes introduced during HoP.

Finally, evaluation of the parameters gestational age, maternal age, birth weight and infant’s sex with the FA profile did not result in any significant correlations or between group differences.

In summary, the presented mid-IR based method bears high potential for studying the effect of environmental parameters or correlations with demographic, anthropometric, or clinical variables on the HM FA profile in clinical studies. Particularly, the short measurement times allow for the analysis of a large number of samples, vastly exceeding the throughput of GC methods.

### 3.5. Greenness Evaluation

**Figure 4:**
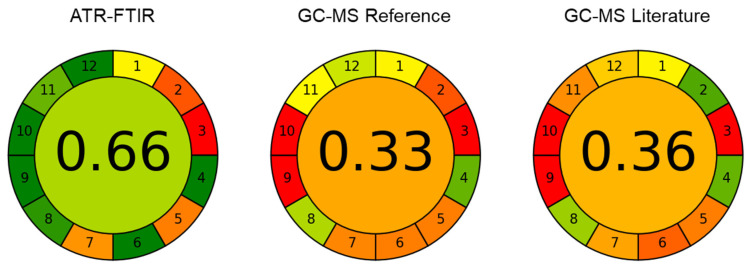
Results of evaluation according to the Analytical GREEnness metric approach for ATR-spectroscopy with solvent-free lipid separation, the applied GC-MS reference method, and a GC-MS method from the literature [15]. The green-yellow-red (i.e., best to worst) colour range represents the performance of each principle.

The presented method based on ATR-FTIR spectroscopy and solvent-free lipid separation was evaluated in terms of accordance with green analytical chemistry (GAC) principles [17]. For this purpose, the previously introduced Analytical GREEnness (AGREE) metric approach [27] was applied, representing a straight-forward assessment tool that considers the twelve principles of GAC (SIGNIFICANCE) [39]. To compare the environmental impact with conventional methods for HM FA determination, ATR-FTIR spectroscopy was juxtaposed with the applied GC-MS reference method and with a GC-MS method from the literature, where the fatty acids are derivatized directly from whole HM [15]. The results are shown in Figure 4 in the form of clock-like graphs comprising of twelve segments that represent the SIGNIFICANCE criteria. Here, the performance of each principle is represented by a green-yellow-red (i.e., best to worst) colour range, whereas the overall performance is expressed by the colour and score (0 = worst, 1 = best) in the middle. The selected options are shown in detail in Table S5. For calculation of the total scores, the individual criteria were equally weighted as indicated by the equal width of the corresponding segments in Figure 4. Based on the total scores of 0.66 for FTIR, 0.33 for the GC-MS reference method, and 0.36 for the GC-MS method from the literature, the presented mid-IR based method shows clear superiority in terms of environmental impact. Compared to the GC methods, its main advantages are high-throughput, reduced energy consumption, the lack of a derivatization step and the absence of hazardous organic solvents. Furthermore, it bears high potential for additional automatization and reduction of waste by replacing the applied disposables such as centrifugation tubes by reusable material. Consequently, the greenness of the presented method based on ATR-FTIR spectroscopy and solvent free-lipid separation can be further improved in the future.

## 4. Conclusions

In this study, the first mid-IR based approach for predicting the individual FA profile of HM was presented. The method comprises a solvent-free lipid separation step for obtaining a representative milk fat fraction, which is then analyzed with ATR-FTIR spectroscopy. A diverse sample set composed of both unprocessed and pasteurized DHM samples was employed for method development and validation. PLS models were established by relating the obtained IR spectra with GC-MS reference concentrations. Compared to a previous study with a limited data set, PLS models for the most relevant FA sum parameters were noticeably improved [26]. Furthermore, excellent results were obtained for the individual FAs—C12:0, C16:0, C18:0, C18:1cis, and C18:2cis. Subsequently, these models were applied to investigate possible correlations between different clinical parameters and the HM FA profile. Here, an increase of MCFA over the course of lactation was observed, whereas LCFA and PUFA decreased, which agreed well with previous studies based on GC-MS data [11]. Finally, the greenness of the presented method was compared to conventional GC-MS methods. Here, beneficial properties such as low energy consumption, lack of a derivatization step and the solvent-free nature resulted in a clear superiority of the mid-IR based method. Consequently, ATR-FTIR combined with solvent-free lipid separation represents a high-throughput, green analytical method allowing for accurate determination of the HM FA composition, which was previously only possible by time-consuming and resource-intensive GC analysis. Finally, the availability of a rapid analytical method for FA profiling is highly relevant in the clinical field to be able to conduct largescale studies within a reasonable timeframe and workload, as the effect of diverse factors on FA composition is still a topic of discussion.

## Figures and Tables

**Table 1:** Results of partial least squares (PLS) regressions for determining relative fatty acid concentrations and sum parameters in g/100 g fat.

		g/100 g Fat						
			Training Set (*n* = 35)				Validation Set (*n* = 15)	
Fatty Acid	LVs	Range	RMSEC	R^2^	RMSECV	R^2^_CV_	RMSEP	R^2^_P_
SAT	6	21–40	0.42	0.99	0.68	0.98	0.54	0.97
MONO	5	29–51	1.1	0.96	1.4	0.94	1.3	0.94
PUFA	4	16–47	1.3	0.96	1.5	0.95	1.4	0.93
UNSAT	6	60–79	0.42	0.99	0.68	0.98	0.54	0.97
SCFA	4	0.40–1.7	0.13	0.78	0.18	0.62	0.15	0.25
MCFA	4	17–36	0.99	0.96	1.3	0.93	0.65	0.94
LCFA	4	63–82	0.87	0.97	1.1	0.95	0.7	0.94
C8:0	2	0.0-0.15	0.022	0.59	0.026	0.52	0.017	0.29
C10:0	4	0.40–1.5	0.12	0.79	0.16	0.63	0.15	0.2
C12:0	6	1.1–7.2	0.18	0.99	0.3	0.96	0.19	0.96
C14:0	6	1.5–7.9	0.26	0.98	0.44	0.94	0.63	0.71
C16:0	6	12–22	0.78	0.92	1.1	0.84	1.1	0.88
C16:1cis	4	0.0–1.2	0.29	0.32	0.42	0.017	0.31	0.19
C18:0	4	1.8–5.9	0.41	0.77	0.55	0.6	0.47	0.75
C18:1cis	6	28–51	1.1	0.96	1.6	0.92	1.4	0.92
C18:2cis	4	16–47	1.2	0.97	1.5	0.95	1.5	0.92

Abbreviations: LVs: latent variables; RMSEC: root mean square error of calibration; R^2^: calibration coefficient of determination; RMSECV: root mean square error of cross-validation; R^2^_CV_: cross-validation coefficient of determination; RMSEP: root mean square error of prediction; R^2^_P_: prediction coefficient of determination; SAT: saturated fatty acids; MONO: monounsaturated fatty acids; PUFA: polyunsaturated fatty acids; UNSAT: unsaturated fatty acids; SCFA: short-chain fatty acids (C10 and lower); MCFA: medium-chain fatty acids (C11-C16); LCFA: long-chain fatty acids (C17 and higher).

## Data Availability

The acquired ATR-FTIR spectra of 50 HM samples can be found online under: https://doi.org/10.5281/zenodo.7064893 (accessed on 30 November 2022). Further data are available on request from the corresponding author.

## Supplementary Materials

The following supporting information can be downloaded at: https://www.mdpi.com/article/10.3390/foods11233906/s1. Figure S1: Heatmap, showing spectral regions included for each PLS model; Formulas for calculating statistical figures of merit; Figure S2: Root mean square error of cross validation (RMSECV, red) and root mean square error of calibration (RMSEC, blue), depending on the number of latent variables for fatty acid sum parameters; Figure S3: Root mean square error of cross validation (RMSECV, red) and root mean square error of calibration (RMSEC, blue), depending on the number of latent variables for individual fatty acids; Figure S4: Measured versus predicted fatty acid content; Figure S5: Loadings plot of FA profiles measured by GC-MS analysis and predicted by ATR-FTIR spectroscopy; Figure S6: Concentration of SAT, MONO, UNSAT, SCFA determined with ATR-FTIR spectroscopy over the course of lactation; Figure S7: Boxplots, comparing relative concentrations of the most important fatty acid sum parameters of donor human milk before and after Holder pasteurization; Table S1: Results of GC-MS analysis for individual FAs in the training set; Table S2: Results of GC-MS analysis for sum parameters in the training set; Table S3: Results of GC-MS analysis for individual FAs in the validation set; Table S4: Results of GC-MS analysis for sum parameters in the validation set; Table S5: Selected options for the Analytical GREEnness evaluation and attributed scores.
