# Supplementary material for: Solvent-Free Lipid Separation and Attenuated Total Reflectance Infrared Spectroscopy for Fast and Green Fatty Acid Profiling of Human Milk
Source: Foods. 2022 Dec 3;11(23):3906. doi: 10.3390/foods11233906 (PMC9741076; doi:10.3390/foods11233906)
Supplement: Supplementary file 1 [file foods-11-03906-s001.zip › foods-2052658-supplementary.pdf]

Supplementary information

Solvent-free Lipid Separation and Attenuated Total Reflectance Infrared Spectroscopy for Fast and Green Fatty Acid Profiling of Human Milk

Christopher K. Akhgar[a], Victoria Ramos-Garcia[b], Vanessa Nürnberger[a,c], Alba Moreno-Giménez[b], Julia Kuligowski[b], Erwin Rosenberg[a], Andreas Schwaighofer[a] and Bernhard Lendl[a*]

[a]Institute of Chemical Technologies and Analytics, Technische Universität Wien, Getreidemarkt 9, 1060 Vienna, Austria

[b]Health Research Institute La Fe, Avenida Fernando Abril Martorell 106, 46026 Valencia, Spain.

[c]Competence Center CHASE GmbH, Altenberger Straße 69, 4040 Linz, Austria

*Corresponding author: Bernhard Lendl, Institute of Chemical Technologies and Analytics, Technische Universität Wien, Getreidemarkt 9, 1060 Vienna, Austria. Email: bernhard.lendl@tuwien.ac.at

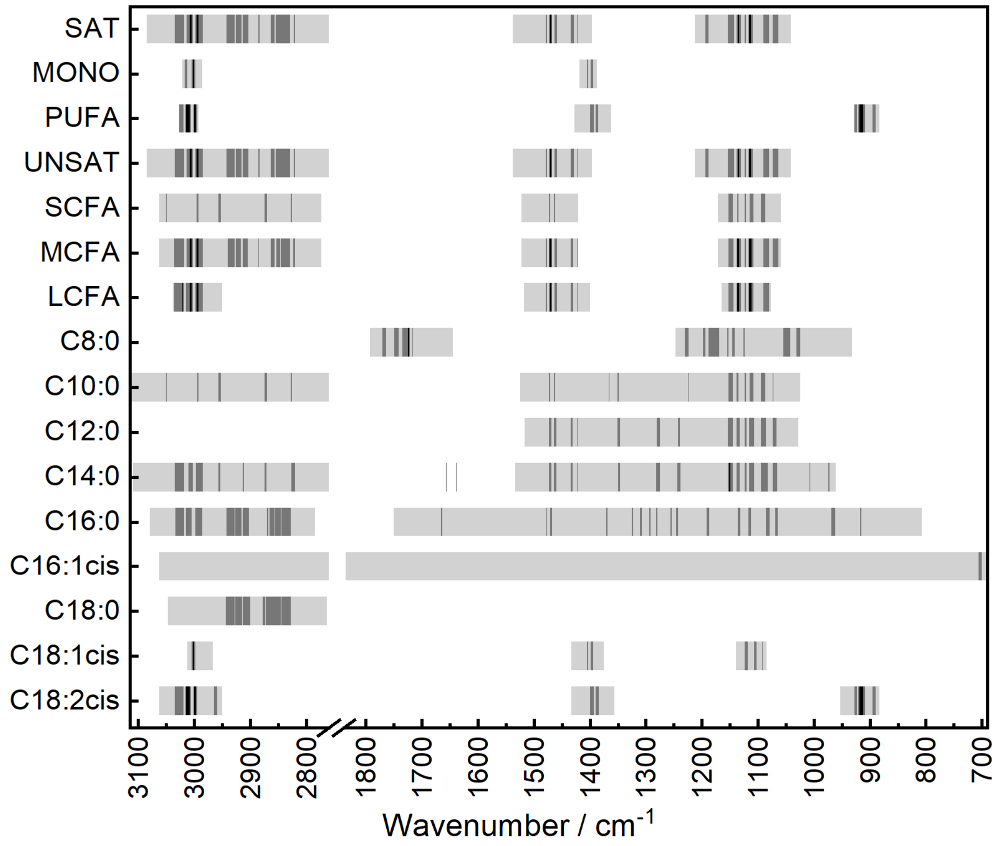

Figure S1. Heatmap, showing spectral regions included for each PLS model in greyscale. Bright grey: selectivity ratio (SR)=0-0.5; dark grey: SR=0.5-5; black: SR=5-15. The spectral range between 1850 and 2750 cm^{-1} was removed due to lack of relevant information. SAT: saturated fatty acids; MONO: monounsaturated fatty acids; PUFA: polyunsaturated fatty acids; UNSAT: unsaturated fatty acids; (C10 and lower); MCFA: medium-chain fatty acids (C11-C16); LCFA: long-chain fatty acids (C17 and higher).

Statistical figures of merit:

- The root mean square error of calibration (RMSEC) was used to estimate the quality of the calibrations and calculated as follows:

$$\mathrm{RMSEC} = \sqrt{\frac{\sum_{i=1}^{N}(y_i - \hat{y}_i)^2}{N}}$$

where N is the number of calibration samples, i.e. 35, y_i are the reference concentrations from GC-MS measurements and $\hat{y}_i$ the results predicted for the calibration set from mid-infrared spectra.

- The calibration coefficient of determination (R^2) was used to determine the fit between GC-MS reference concentrations and the results predicted for the calibration set from mid-infrared spectra and calculated as follows:

$$\mathrm{R}^2 = 1 - \frac{\sum_{i=1}^{N}(\mathrm{y_i} - \hat{\mathrm{y}}_\mathrm{i})^2}{\sum_{i=1}^{N}(\mathrm{y_i} - \bar{\mathrm{y}}_\mathrm{i})^2}$$

where N is the number of calibration samples, i.e. 35, y_i are the reference concentrations from GC-MS measurements, $\hat{y}_i$ are the results predicted for the calibration set from mid-infrared spectra and $\bar{y}_i$ is the mean value of the reference results..

- The root mean square error of cross-validation (RMSECV) was used to estimate the predictive ability of the models and to select an appropriate number of latent variables and calculated as follows:

$$\mathrm{RMSECV} = \sqrt{\frac{\sum_{i=1}^{N}(y_i - \hat{y}_i)^2}{N}}$$

where N is the number of calibration samples, i.e. 35, y_i are the reference concentrations from GC-MS measurements and $\hat{y}_i$ are the mid-infrared results predicted via cross-validation.

- The cross-validation coefficient of determination (R^2_{CV}) was used to determine the fit between GC-MS reference concentrations and the concentrations predicted via cross-validation and calculated as follows:

$$\mathrm{R^2}_{\mathrm{CV}} = 1 - \frac{\sum_{i=1}^{N}(y_i - \hat{y}_i)^2}{\sum_{i=1}^{N}(y_i - \bar{y}_i)^2}$$

where N is the number of calibration samples, i.e. 35, y_i are the reference concentrations from GC-MS measurements, $\hat{y}_i$ are the mid-infrared results predicted via cross-validation and $\bar{y}_i$ is the mean value of the reference results.

- The root mean square error of prediction (RMSEP) was used to evaluate the ability of the models for predicting concentrations of an independent, external validation set and calculated as follows:

$$\mathrm{RMSEP} = \sqrt{\frac{\sum_{i=1}^{N}(y_i - \hat{y}_i)^2}{N}}$$

where N is the number of external validation samples, i.e. 15, y_i are the reference concentrations from GC-MS measurements and $\hat{y}_i$ are the results predicted for the external validation set.

- The prediction coefficient of determination (R^2_P) was used to determine the fit between GC-MS reference concentrations and the results predicted from the external validation set and calculated as follows:

$$\mathrm{R}^2{}_\mathrm{P} = 1 - \frac{\sum_{i=1}^{N}(y_i - \hat{y}_i)^2}{\sum_{i=1}^{N}(y_i - \bar{y}_i)^2}$$

where N is the number of external validation samples, i.e. 15, y_i are the reference concentrations from GC-MS measurements, $\hat{y}_i$ are the results predicted for the external validation set and $\bar{y}_i$ is the mean value of the reference results.

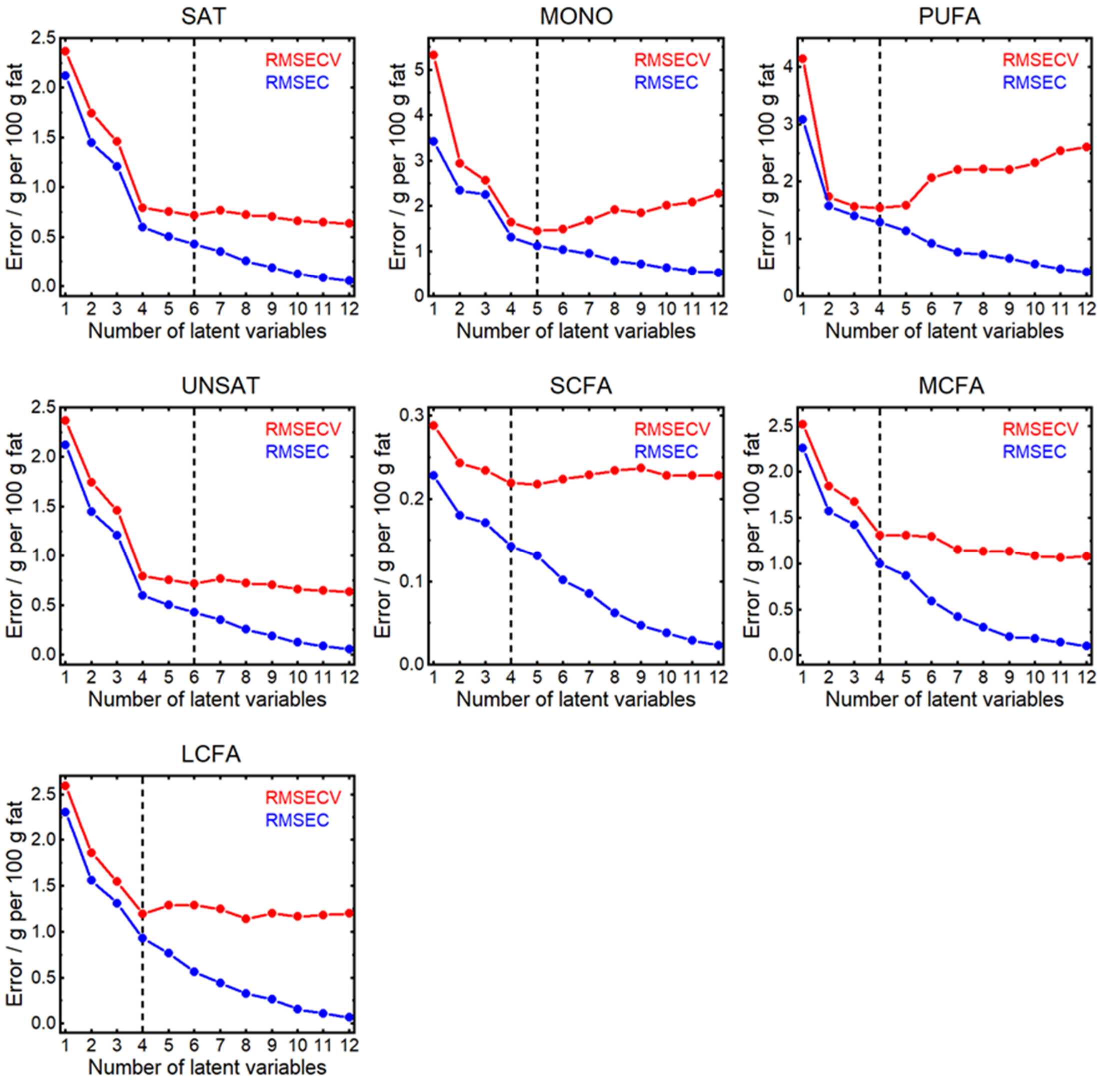

Figure S2. Root mean square error of cross validation (RMSECV, red) and root mean square error of calibration (RMSEC, blue), depending on the number of latent variables for fatty acid sum parameters. The dashed lines indicate the selected number of latent variables for each individual PLS model.

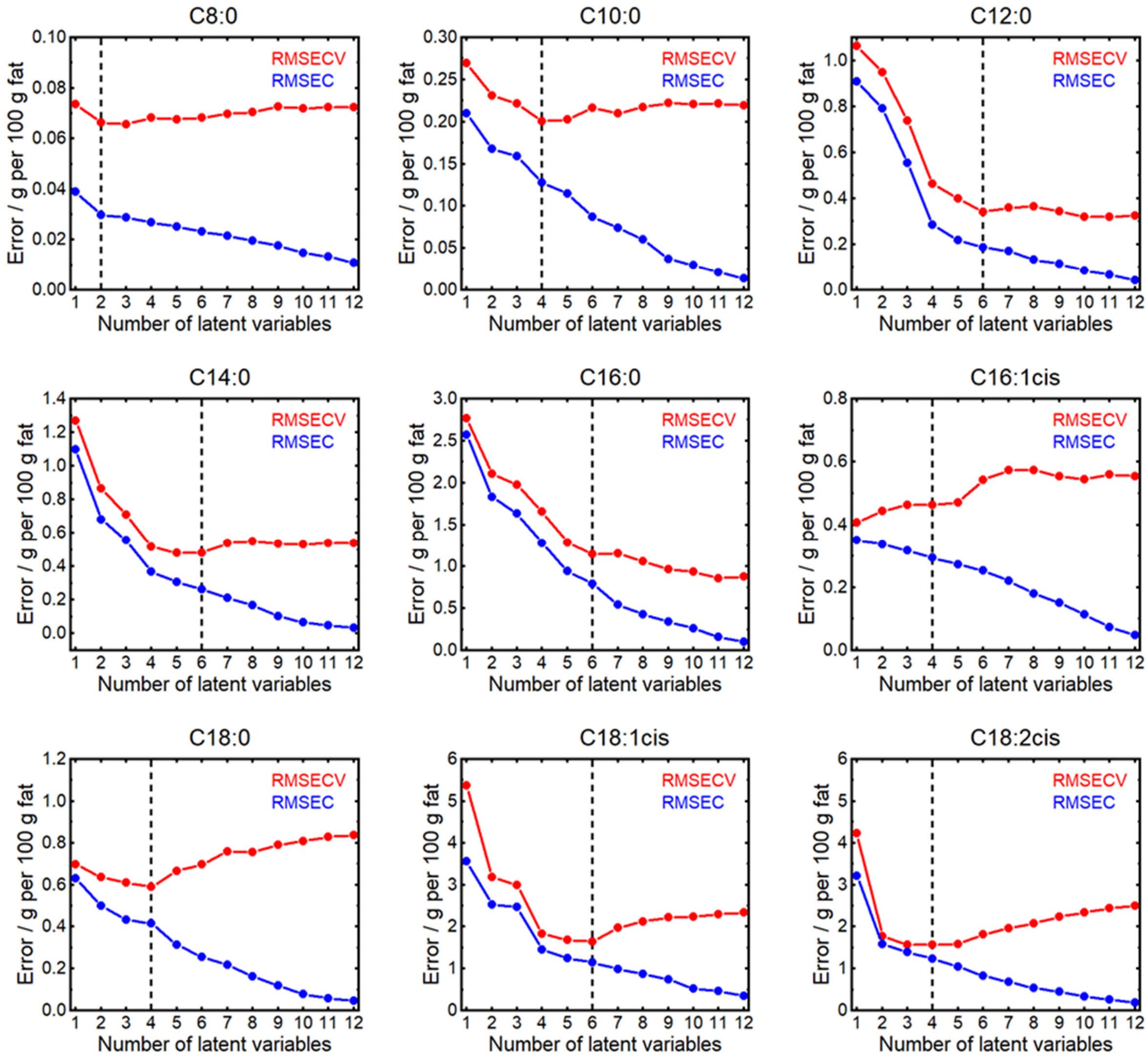

Figure S3. Root mean square error of cross validation (RMSECV, red) and root mean square error of calibration (RMSEC, blue), depending on the number of latent variables for individual fatty acids. The dashed lines indicate the selected number of latent variables for each individual PLS model.

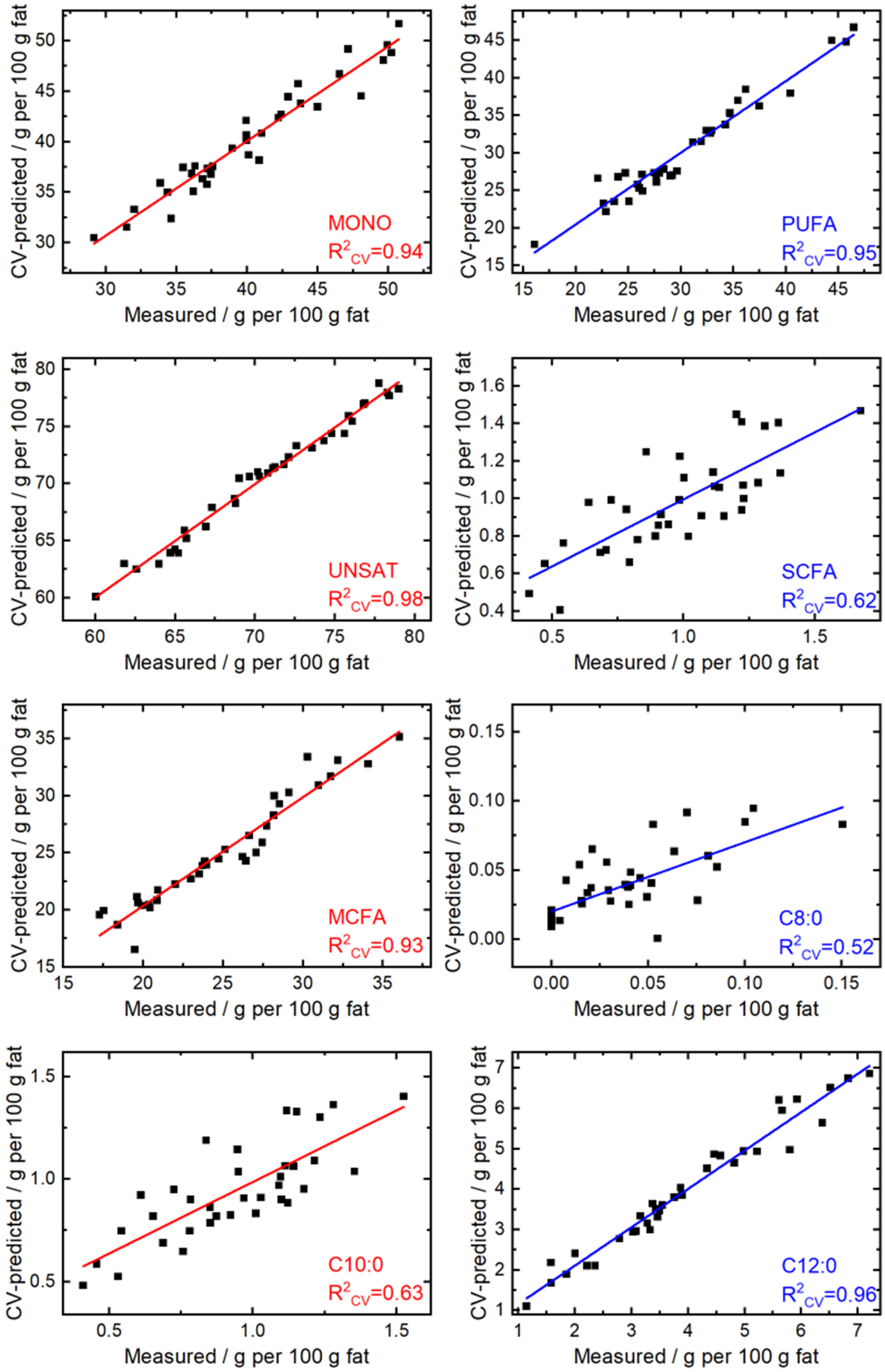

Figure S4. Measured (GC-MS) versus predicted (ATR-FTIR, cross-validation) fatty acid content.

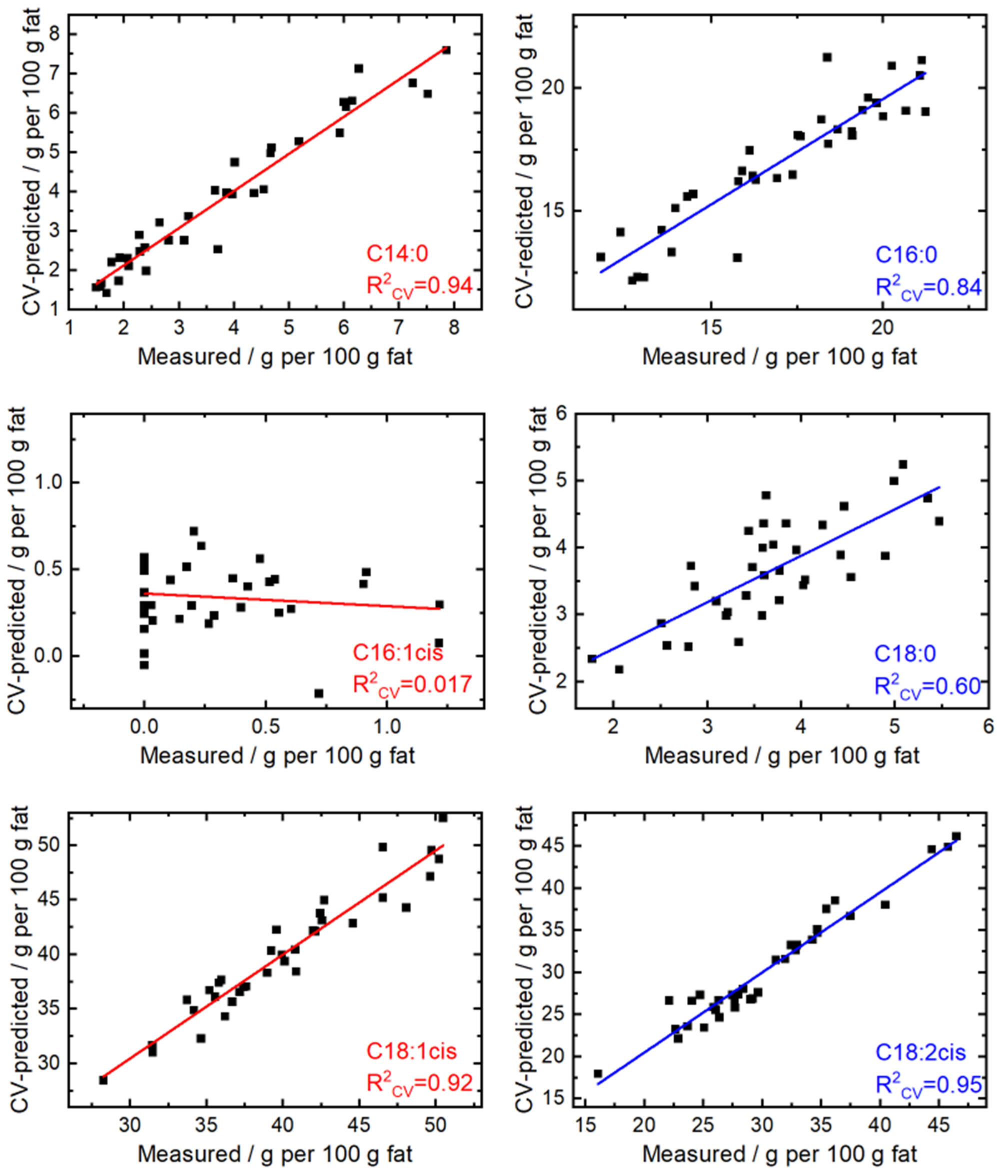

Figure S4 continuation. Measured (GC-MS) versus predicted (ATR-FTIR, cross-validation) fatty acid content.

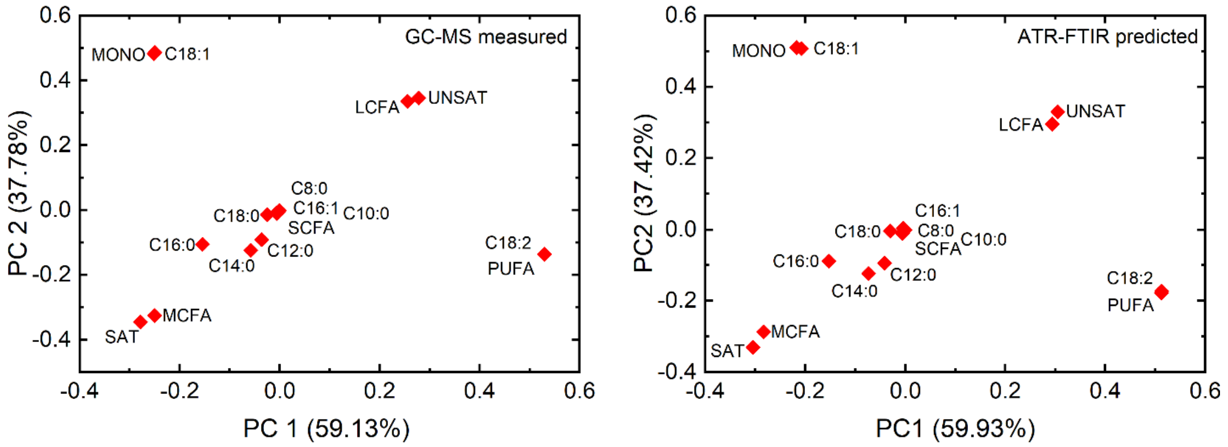

Figure S5. Loadings plot of FA profiles measured by GC-MS analysis (left) and predicted by ATR-FTIR spectroscopy (right).

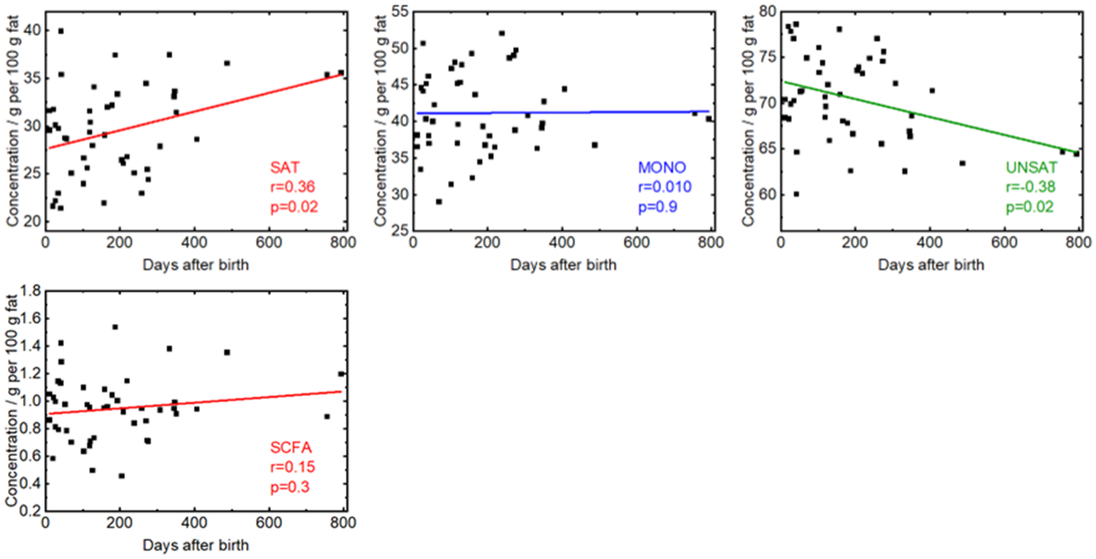

Figure S6. Concentration of saturated fatty acids (SAT), monounsaturated fatty acids (MONO), unsaturated fatty acids (UNSAT) and short-chain fatty acids (SCFA), determined with ATR-FTIR spectroscopy over the course of lactation.

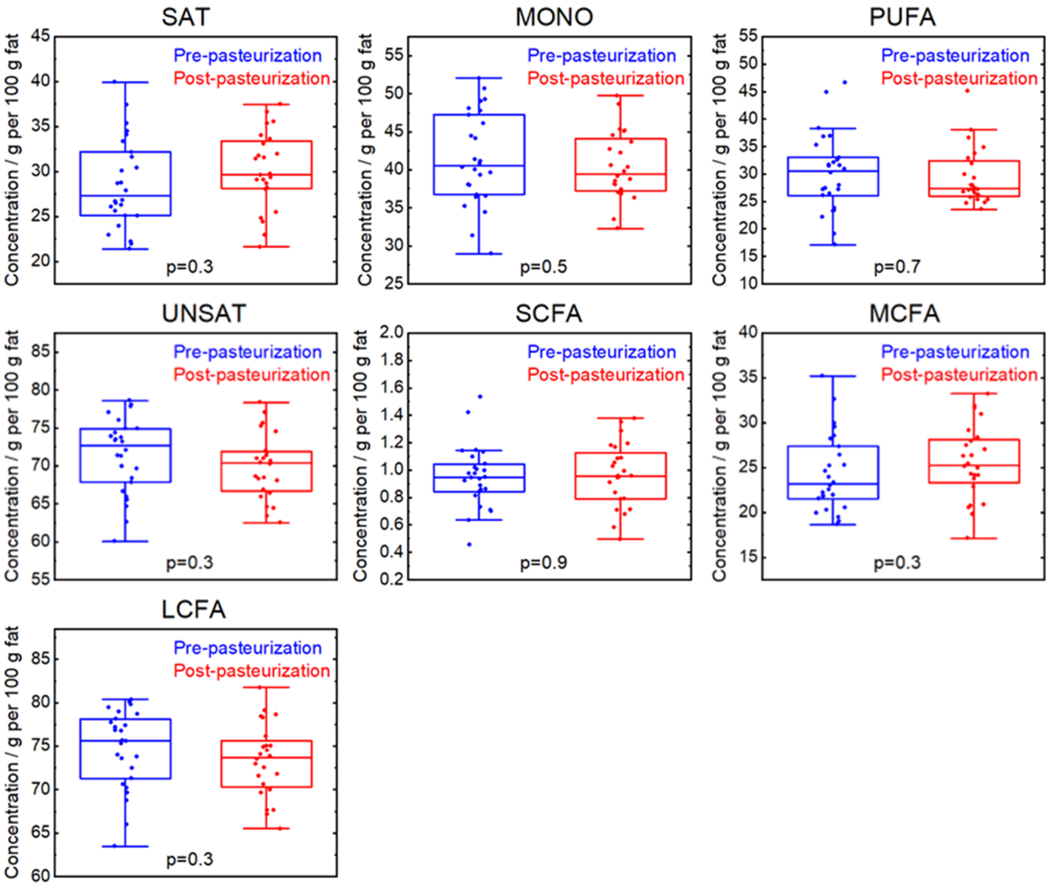

Figure S7. Boxplots, comparing relative concentrations of the most important fatty acid sum parameters of donor human milk before (blue) and after (red) Holder pasteurization, including p-values from two-sample t-tests.

Table S1. Results of GC-MS analysis for individual FAs (in g/100 g fat) in the training set.

Sample code	C8:0	C10:0	C12:0	C14:0	C16:0	C16:1 cis	C18:0	C18:1 c	C18:2 c
HM HoP 14	0.0	1.2	5.9	6.1	18.2	0.0	3.3	36.2	29.0
HM 31	0.0	0.9	2.8	2.6	21.2	0.4	3.4	44.6	24.0
HM HoP 22	0.0	0.4	1.1	1.6	19.8	0.5	5.0	42.4	29.2
HM HoP 9	0.0	0.8	2.2	2.1	19.4	0.0	5.3	48.1	22.1
HM 4	0.0	0.8	4.5	5.9	18.4	0.4	5.5	39.6	25.1
HM HoP 21	0.1	1.2	4.8	4.0	16.3	0.0	3.4	37.4	32.8
HM HoP 26	0.0	0.8	2.4	2.3	20.7	0.9	4.2	42.7	26.0
HM 5	0.0	0.9	3.5	3.9	19.1	0.2	3.8	36.7	31.9
HM HoP 28	0.1	1.2	6.8	7.9	19.1	0.3	3.1	35.2	26.3
HM HoP 12	0.1	0.9	4.6	4.4	15.8	0.0	3.5	34.6	36.2
HM HoP 11	0.0	0.7	3.1	2.1	14.5	0.0	4.9	40.1	34.7
HM 24	0.1	1.1	4.3	3.2	12.9	0.0	2.6	31.5	44.4
HM 7	0.0	0.7	1.8	1.9	16.2	0.9	3.8	28.2	46.4
HM HoP 20	0.0	0.6	3.3	3.7	20.0	0.4	3.6	42.0	26.3
HM 11	0.0	0.7	3.8	4.5	16.9	1.2	2.8	42.6	27.5
HM HoP 1	0.0	0.5	2.0	1.8	15.9	0.0	3.7	50.2	25.9
HM 16	0.1	1.3	6.5	7.2	21.1	1.2	3.8	36.0	22.9
HM HoP 5	0.0	0.5	1.6	1.6	15.8	0.6	2.9	31.4	45.7
HM HoP 3	0.0	1.1	5.2	4.7	14.0	0.0	3.2	37.6	34.2
HM HoP 4	0.0	1.4	6.4	6.0	16.1	0.0	4.5	40.9	24.7
HM HoP 31	0.0	1.0	3.9	4.0	20.3	0.0	3.9	39.0	28.0
HM 8	0.0	1.1	5.8	4.7	13.0	0.0	2.8	37.2	35.4
HM 23	0.0	1.0	3.0	1.7	12.4	0.2	3.6	49.7	28.4
HM 13	0.1	0.9	3.4	3.1	14.3	0.1	4.0	33.7	40.4
HM 26	0.2	1.5	7.2	6.3	18.7	0.0	3.6	39.9	22.6
HM HoP 15	0.0	0.9	5.6	7.5	17.6	0.2	3.6	40.8	23.7
HM 3	0.1	1.1	3.5	2.3	17.5	0.7	4.4	39.2	31.2
HM 18	0.0	1.1	5.0	5.2	17.4	0.2	4.0	34.2	32.9
HM HoP 24	0.1	1.1	5.7	6.0	19.6	0.5	3.6	35.8	27.7
HM 21	0.0	0.8	3.3	2.4	12.7	0.0	1.8	46.5	32.4
HM 9	0.0	0.5	1.6	1.5	18.4	0.5	4.5	35.5	37.5
HM 14	0.0	1.0	3.4	3.7	21.1	0.0	5.1	49.6	16.1
HM 20	0.1	1.2	3.9	2.4	13.6	0.1	2.1	42.1	34.6
HM HoP 27	0.0	0.9	3.2	2.8	13.9	0.6	2.5	46.5	29.6
HM 29	0.1	1.1	3.5	1.9	11.8	0.3	3.2	50.5	27.7

Table S2. Results of GC-MS analysis for sum parameters (in g/100 g fat) in the training set.

Sample code	SAT	MONO	PUFA	UNSAT	SCFA	MCFA	LCFA
HM HoP 14	34.8	36.2	29.0	65.2	1.2	30.3	68.5
HM 31	31.0	45.0	24.0	69.0	0.9	27.1	72.0
HM HoP 22	27.9	42.9	29.2	72.1	0.4	23.0	76.6
HM HoP 9	29.8	48.1	22.1	70.2	0.8	23.7	75.5
HM 4	35.0	39.9	25.1	65.0	0.8	29.1	70.1
HM HoP 21	29.7	37.4	32.8	70.3	1.2	25.1	73.7
HM HoP 26	30.4	43.6	26.0	69.6	0.8	26.2	73.0
HM 5	31.2	36.8	31.9	68.8	0.9	26.6	72.4
HM HoP 28	38.2	35.5	26.3	61.8	1.3	34.1	64.6
HM HoP 12	29.2	34.6	36.2	70.8	1.0	24.7	74.3
HM HoP 11	25.2	40.1	34.7	74.8	0.7	19.6	79.7
HM 24	24.2	31.5	44.4	75.8	1.2	20.4	78.4
HM 7	24.4	29.2	46.4	75.6	0.7	20.9	78.5
HM HoP 20	31.3	42.4	26.3	68.7	0.6	27.5	71.9
HM 11	28.8	43.8	27.5	71.2	0.7	26.4	72.9
HM HoP 1	23.9	50.2	25.9	76.1	0.5	19.7	79.8
HM 16	40.0	37.2	22.9	60.0	1.4	36.0	62.6
HM HoP 5	22.3	32.0	45.7	77.7	0.5	19.5	80.1
HM HoP 3	28.2	37.6	34.2	71.8	1.1	23.9	75.0
HM HoP 4	34.4	40.9	24.7	65.6	1.4	28.5	70.1
HM HoP 31	33.1	39.0	28.0	66.9	1.0	28.2	70.9
HM 8	27.4	37.2	35.4	72.6	1.1	23.5	75.4
HM 23	21.7	49.9	28.4	78.3	1.1	17.3	81.7
HM 13	25.7	33.9	40.4	74.3	0.9	20.9	78.2
HM 26	37.4	39.9	22.6	62.6	1.7	32.2	66.1
HM HoP 15	35.3	41.0	23.7	64.7	1.0	31.0	68.1
HM 3	28.9	39.9	31.2	71.1	1.2	24.0	74.8
HM 18	32.7	34.4	32.9	67.3	1.1	27.7	71.1
HM HoP 24	36.0	36.3	27.7	64.0	1.2	31.7	67.0
HM 21	21.0	46.5	32.4	79.0	0.9	18.4	80.8
HM 9	26.4	36.1	37.5	73.6	0.5	22.0	77.5
HM 14	34.3	49.6	16.1	65.7	1.0	28.2	70.8
HM 20	23.1	42.2	34.6	76.9	1.3	19.9	78.8
HM HoP 27	23.2	47.1	29.6	76.8	0.9	20.4	78.7
HM 29	21.6	50.7	27.7	78.4	1.2	17.5	81.3

Table S3. Results of GC-MS analysis for individual FAs (in g/100 g fat) in the validation set.

Sample Code	C8:0	C10:0	C12:0	C14:0	C16:0	C16:1 cis	C18:0	C18:1 c	C18:2 c
HM HoP 6	0.0	1.1	3.0	2.7	18.8	0.5	3.9	40.4	29.7
HM HoP 29	0.1	1.0	3.0	3.1	20.6	0.4	3.9	42.4	25.5
HM 10	0.0	1.0	3.2	4.7	14.3	0.0	3.3	40.4	33.4
HM 28	0.0	0.5	2.8	2.7	22.0	0.5	5.9	45.8	19.5
HM 17	0.0	0.9	3.6	3.0	16.9	0.0	3.5	39.0	33.1
HM HoP 16	0.0	0.8	5.2	5.9	15.2	0.0	2.8	40.4	29.5
HM HoP 2	0.0	1.0	4.5	4.2	16.9	0.0	4.4	35.6	33.6
HM 25	0.0	0.8	2.7	2.3	17.1	0.7	3.3	49.9	23.1
HM 15	0.0	0.9	4.9	4.9	17.7	0.0	5.2	36.5	29.9
HM HoP 13	0.0	1.0	4.1	4.1	20.0	0.1	3.9	38.0	28.7
HM HoP 10	0.0	1.0	3.4	2.8	13.8	0.0	3.6	38.0	37.7
HM HoP 23	0.0	0.8	4.6	3.7	19.6	0.0	2.7	46.3	22.1
HM 1	0.0	1.0	3.7	2.8	14.7	0.0	3.4	47.6	26.7
HM 12	0.0	1.0	2.5	2.0	19.7	0.6	5.0	37.9	31.4
HM 2	0.0	0.7	1.1	1.5	20.0	0.0	3.9	49.4	23.5

Table S4. Results of GC-MS analysis for sum parameters (in g/100 g fat) in the validation set.

Sample code	SAT	MONO	PUFA	UNSAT	SCFA	MCFA	LCFA
HM HoP 6	29.5	40.8	29.7	70.5	1.0	24.9	74.0
HM HoP 29	31.7	42.8	25.5	68.3	1.0	27.2	71.8
HM 10	26.1	40.4	33.4	73.9	0.6	22.3	77.2
HM 28	34.3	46.3	19.5	65.7	0.9	28.0	71.1
HM 17	27.9	39.0	33.1	72.1	0.8	23.6	75.7
HM HoP 16	30.1	40.4	29.5	69.9	1.1	26.2	72.7
HM HoP 2	30.9	35.6	33.6	69.1	0.8	25.7	73.5
HM 25	26.3	50.6	23.1	73.7	0.9	22.9	76.2
HM 15	33.7	36.5	29.9	66.3	1.0	27.5	71.5
HM HoP 13	33.1	38.1	28.7	66.9	1.0	28.3	70.7
HM HoP 10	24.4	38.0	37.7	75.6	0.8	20.0	79.2
HM HoP 23	31.6	46.3	22.1	68.4	1.1	27.9	71.1
HM 1	25.6	47.6	26.7	74.4	1.0	21.2	77.8
HM 12	30.1	38.5	31.4	69.9	0.8	24.9	74.3
HM 2	27.1	49.4	23.5	72.9	0.5	22.7	76.8

Table S5. Selected options for the Analytical GREEnness evaluation and attributed scores according to (Pena-Pereira, Wojnowski, & Tobiszewski, 2020).

Parameter	ATR		GC Reference		GC Literature	
	Selected option	Score	Selected option	Score	Selected option	Score
1. Sampling procedure	Off-line analysis	0.48	Off-line analysis	0.48	Off-line analysis	0.48
2. Amount of sample in g or mL	30	0.17	30	0.17	0.25	0.85
3. Position of analytical device	Off-line	0.0	Off-line	0.0	Off-line	0.0
4. Sample preparation steps	3 or fewer	1.0	4	0.8	4	0.8
5. Automation, miniaturization	Semi-automatic, non-miniaturized	0.25	Semi-automatic, non-miniaturized	0.25	Semi-automatic, non-miniaturized	0.25
6. Derivatization	None	1.0	Cas: 17287-03-5	0.25	Cas: 13228-87-6	0.19
7. Amount of waste in g or mL	20	0.29	25	0.26	16	0.32
8. Number of analytes per run, sample throughput per hour	9, 6	0.92	37, 0.5	0.66	36, 0.6	0.69
9. Most-energy intensive	FTIR	1.0	GC-MS	0.0	GC-MS	0.0
10. Type of reagents	No reagents	1.0	Not bio-based	0.0	Not bio-based	0.0
11. Toxic reagents or solvents in g or mL	0.1	0.8	1.1	0.49	5.6	0.28
12. Threats	None	1.0	Flammable	0.6	Toxic to aquatic life, flammable, corrosive	0.4

References

Pena-Pereira, F., Wojnowski, W., & Tobiszewski, M. (2020). AGREE—Analytical GREEnness Metric Approach and Software. *Analytical Chemistry, 92*(14), 10076-10082. https://doi.org/10.1021/acs.analchem.0c01887
